# Recognition of Proteins by Metal Chelation-Based Fluorescent Probes in Cells

**DOI:** 10.3389/fchem.2019.00560

**Published:** 2019-08-09

**Authors:** Nan Jiang, Hongyan Li, Hongzhe Sun

**Affiliations:** Department of Chemistry, The University of Hong Kong, Hong Kong, China

**Keywords:** His-tag, metallomics, metalloproteomics, molecular imaging, thiol-reaction

## Abstract

Fluorescent probes such as thiol-reactive and Ni^2+^-nitrilotriacetate (NTA) based probes provide a powerful toolbox for real-time visualization of a protein and a proteome in living cells. Herein, we first went through basic principles and applications of thiol-reactive based probes in protein imaging and recognition. We then summarize a family of metal-NTA based fluorescence probes in the visualization of His_6_-tagged protein and identification of metalloproteins at proteome-wide scale. The pros and cons of the probes, as well as ways to optimize them, are discussed.

## Introduction

In the past decades, small molecule-based probes have been extensively used in protein labeling, allowing *in situ* studies of function, subcellular localization, and dynamics of the proteins of interest (POIs). Because of their relatively small sizes, these probes have less perturbation on the investigated proteins (Giepmans et al., 2006; Sletten and Bertozzi, 2009). Particularly, the small molecule-based tagging system has received growing attention, the POI is genetically fused with a short peptide of specific motifs, which is subsequently recognized by the probes. The recognition of POI by the probe either through chemical reaction or metal chelation usually results in fluorescent responses, which is applicable for biological imaging and quantification. Metal-chelation labeling of a protein is particularly attractive owing to its simplicity and high specificity. Pioneered by Griffin et al. (1998), a family of biarsenical probes have been developed, which specifically recognize a tetracysteine tag. Given that the (histidine)_6_-tag is more widely used in protein purification, enormous efforts have been made to develop fluorescence probes to selectively recognize the His_6_-tag (Kapanidis et al., 2001; Hauser and Tsien, 2007). These probes have been further utilized to label endogenous metal-binding proteins, providing a useful tool for metalloproteomics (Zhang et al., 2015; Jiang et al., 2018).

In this mini-review, we focus on metal chelation-based protein labeling, typically on the development of fluorescence probes that specifically recognize tetra-cysteine- and His_6_-tags in live cells. We highlight the optimization of the probes and their utilities in various systems. We also discuss the optimizations of fluorophores and molecule delivery for probes in tracking intracellular proteins.

## The Thiol-Reactive Fluorescent Probes

### Protein Recognition Based on Thiol Reaction

**Figure 1:**
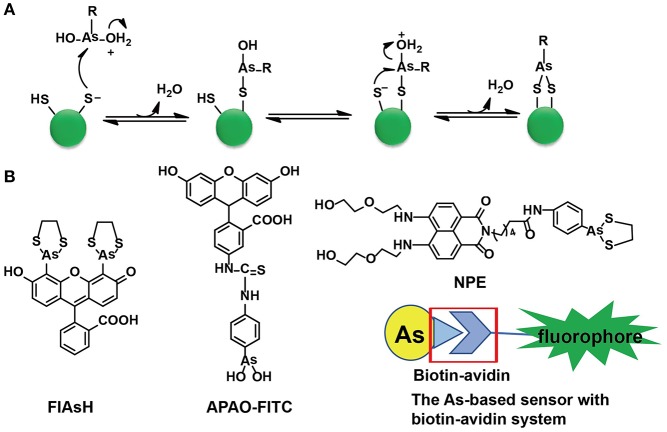
The As(III)-based thiol-reactive fluorescent probes. **(A)** The recognition mechanism of As(III) binding toward two closely spaced cysteines. The buffer action can make several RAs(OH)_2_ molecules to be protonated at the hydroxyl oxygen (R stands for any other chemical group). The attack of a thiolate anion on As(III) can release a water molecule. Then a proton transfer between the other thiolate anion and the hydroxyl oxygen is induced by the buffer action whilst the attack of this thiolate anion on As(III) will proceed an intramolecular displacement of another water molecule. **(B)** The structures of typical As(III)-based probes designed by different strategies. **(A)** was adapted from Gasser (2014) Copyright 2014 Wiley.

Cysteine residues are quite abundant and vital in protein structures to link two cysteine-containing peptides. Among 3,758 identified human proteins, more than 15,000 cysteine residues were reported (Gasser, 2014). The high abundance of cysteine residues has inspired scientists to develop strategies to label proteins via these sites. Although both As(III) and Sb(III) have high affinity to cysteine residues, only As(III) has been selected in the development of sensors. As-S coordination bonds are generated through thiol exchange, enabling As(III) to recognize cysteine residues. Compared with the liner structures created by As(III) and monothiols, the ring structures consisting of As(III) and two closely spaced thiols appear to be much more stable due to the factor of entropy (Stocken and Thompson, 1946). This explains why the formation of dithiol-reactive As(III) complexes is the main trend and the distance between the two cysteine residues was estimated to be 3–4 Å (Adams et al., 1990; Bhattacharjee and Rosen, 1996). When As(III) meets di-vicinal cysteine residues, chelation of As(III) to cysteines is achieved (Figure 1A).

### Designs of Arsenic-Based Fluorescent Probes

A family of arsenic(III)-based probes was designed and further modified for *in situ* visualizations of proteins with genetically fused tetra-cysteine residues. Roger Tsien et al. firstly reported a membrane-permeable and non-fluorescent As(III)-complex, i.e., FLAsH-EDT_2_ (EDT = ethanedithiol), to site-specifically recognize proteins containing four cysteines at the *i, i* + 1, *i* + 4, and *i* + 5 positions of an α-helix in 1998 (Griffin et al., 1998). A few years later, they found that four cysteines occurring in hairpin conformation were more preferred than the α-helix. Subsequently, a library of FLAsH-EDT_2_ analogs was developed and ReAsH-EDT_2_ has been regarded as the most suitable one to target a hairpin structure (Adams et al., 2002).

Both FLAsH-EDT_2_ and ReAsH-EDT_2_ are turn-on fluorescent probes as the 1,3,2-dithiarsolane (EDT) results in weak fluorescence before thiol-reaction with cysteines. Such a benefit was inherited in the design of other As-based probes, NPE, and CTNPE. Due to the structural features of vicinal-dithiol-containing proteins (VDPs), only one As(III) was kept for protein recognition, whilst two biocompatible diglycol amine groups were integrated into these probes to improve their water solubility and biological compatibility (Huang et al., 2011). Based on this work, a family of probes with various linkers (6-aminocaproic acid, succinic acid, and piperazine) were designed to detect VDPs in diverse conditions (different pH response, hydrophobicity, etc.; Huang et al., 2013). Later, the same group designed the first generation of radiometric fluorescent probe for VDPs, VTAF. This probe can be used to quantify the VDPs via the readout of the radiometric fluorescence signal with high selectivity (Huang et al., 2014). Besides these EDT-containing molecules, a similar As(III)-based probe, APAO-FITC, was synthesized with only one As-based moiety with the EDT being replaced by two hydroxyl groups (-OH). APAO-FITC has been shown to label native As-binding proteins without strong disturbance on their secondary structures (Femia et al., 2012). To expand the toolbox of As(III)-based probes, the biotin-avidin system was then taken into consideration while the As(III)-binding proteins could be analyzed by the combination of proteome microarray assay with biotin-conjugated As, i.e., Biotin-As. Given the high affinity of biotin toward avidin, the proteins distinguished by Biotin-As via thiol reaction quickly bind to the avidin-fluorophore to turn on the fluorescence (Figure 1B; Zhang et al., 2015).

### Targeting Cysteine in Diverse Systems

The first generation of As(III)-based fluorescent probes, FLAsH-EDT_2_ and ReAsH-EDT_2_, mainly target proteins with genetically fused tetracysteine tag since this motif seems to be rare among native proteins (Femia et al., 2012). Moreover, two As-based moieties of the probes result in stable protein labeling, and these two probes work successfully in the visualization of intracellular proteins as well as in affinity chromatography to track arsenic-binding proteins (Griffin et al., 1998). Importantly, FLAsH-EDT_2_ and ReAsH-EDT_2_ have also been used to study the protein-protein interactions and protein folding by Luedtke et al. (2007). The tetracysteine tag was introduced to several vital biomarkers, e.g., avian pancreatic polypeptide (aPP), to estimate the protein mis-folding in the development of brain diseases by monitoring the fluorescent change of ReAsH-EDT_2_.

Besides those tetra-cysteine fused proteins, the thiol-groups of native proteins play a principal role in the balance of intracellular redox environment (Ying et al., 2007), whilst those proteins usually have the dithiol-motif such as -CX_*n*_C- rather than tetracysteine. Acting as the reductive end of this oxidation-reduction network, VDPs can be labeled by NPE both *in vitro* and *in vivo*. Staining of NPE finally lit up the VDPs in live cells without invasiveness (Huang et al., 2011). The analysis of As(III)-proteomes was achieved (Zhang et al., 2015) and the Biotin-As(III) serves as the As(III)-protein sensor following the protein separation by human proteome microarrays. In total 360 As(III)-binding proteins were reported and most of them were involved in the pathway of glycolysis. Subsequently, hexokinase-2 (HK2) was further studied by biochemical and metabolomics analysis. Consequently, they validated the identified As(III)-binding proteins which could be potential drug targets.

## Design of Metal-NTA Based Fluorescent Probes

### His_6_-Ni^2+^-NTA System for Imaging Proteins

The first report on using the His_6_-Ni^2+^-NTA system for protein purification was dated back to 1975 (Porath et al., 1975). It became a routine method for purification of His_6_-tagged proteins (Arnau et al., 2006). The wide usage of the His_6_-Ni^2+^-NTA system inspired scientists to visualize proteins by conjugating signaling groups, usually fluorophores. The first fluorescent modification on Ni-NTA was made by Kapanidis et al. (2001) and Katayama in 2001 (Amano et al., 2002). Subsequently, this type of probe was utilized to label membrane His-tagged proteins by Guignet et al. (2004) and Zenmyo et al. (2019).

**Figure 2:**
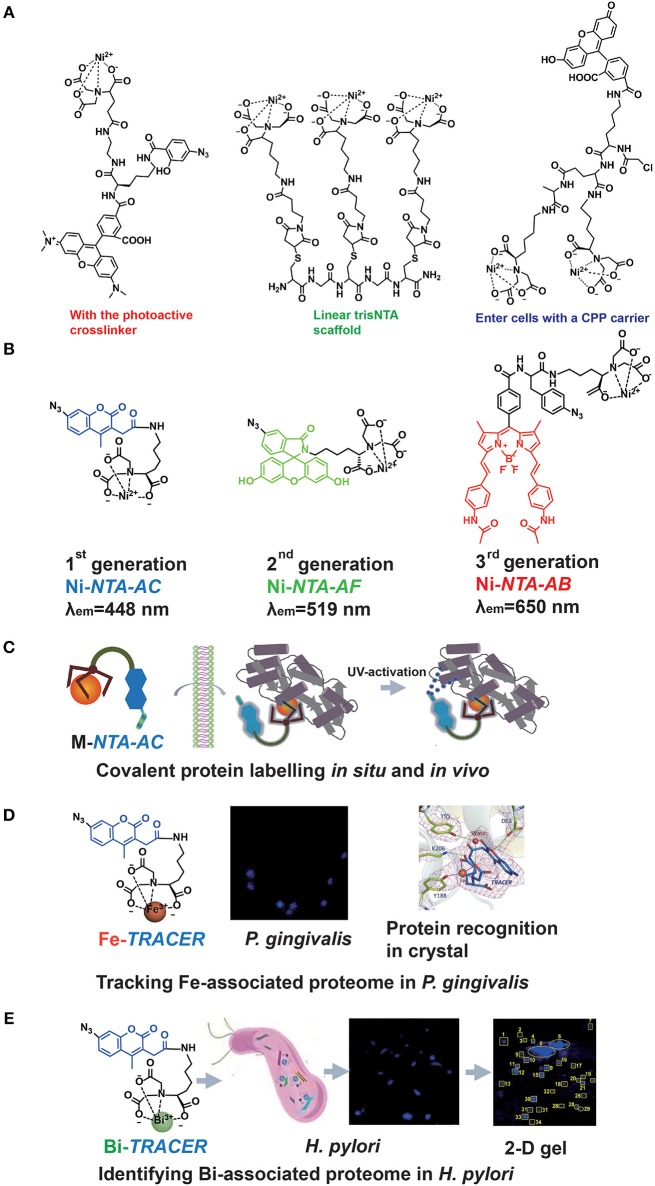
The probe family based upon NTA-metal coordination with their applications in protein tracking. Typical Ni-NTA-based probes were presented in **(A)** whilst our three generations of homemade probes were listed as **(B)**, including **Ni-*NTA-AC***, **Ni-*NTA-AF***, and **Ni-*NTA-AB***. Then the explorations for metal-associated proteomes in diverse system by using **M-*TRACER*** were presented as **(C)** the covalent labeling ability of **M-*TRACER*** toward intracellular proteins with UV-activation, **(D)** Fe-associated proteome tracked by **Fe-*TRACER*** in *P. gingivalis* and the protein-binding model released by X-ray crystallography, **(E)** Bi-associated proteome labeled by **Bi-*TRACER*** in *H. pylori*. **(C,D)** were reproduced from Jiang et al. (2018) with the permission of The Royal Society of Chemistry. **(E)** was reproduced from Wang et al. (2017) with the permission of The Royal Society of Chemistry.

The major drawback of the Ni-NTA system appeared to be the relatively low affinity of NTA toward Ni^2+^ with a dissociation constant of 1–20 μM (Soh, 2008). Hence, the multivalent tactic by conjugating more than one Ni-NTA moiety was developed (Guignet et al., 2004; Soh, 2008; Jing and Cornish, 2011). Very recently, R. Tampé and his co-workers designed linear, dendritic, and cyclic *tris*NTA scaffolds and compared their labeling abilities toward His-tagged proteins in fixed cells. Nevertheless, these multi-NTA probes can be hardly used in imaging of intracellular proteins owing to larger molecule size, which limited them to enter cells (Gatterdam et al., 2018). The other disadvantage is the fluorescent quenching triggered by paramagnetic Ni^2+^ in bio-imaging. By introducing NTA at the 6-position in the fluorescein ring, Lippard et al. developed a new Ni-NTA based probe with slighter fluorescent quenching, due to the molecular spatial conformation upon Ni^2+^ chelation (Goldsmith et al., 2006). To strengthen the protein binding, photoactive crosslinkers were firstly integrated by Auer et al. into the probes to form a stable covalent bond upon UV-irradiation (Hintersteiner et al., 2008). Hamachi et al. also synthesized a nucleophilic reaction-based Ni-NTA probe to label His-tagged proteins covalently (Uchinomiya et al., 2009). Nevertheless, almost all those probes suffer exclusively poor membrane permeability while a cell penetrating peptide (CPP) carrier was used to address this issue (Uchinomiya et al., 2013; Figure 2A). The usage of Ni-NTA was even combined with Fe_3_O_4_ nanoparticles to increase the adsorption efficiency (Guo et al., 2018).

Recently, a new family of Ni-NTA based fluorescent probes, which can rapidly enter cells to label His_6_-tagged protein covalently, was developed. The first probe, **Ni-*NTA-AC***, consists of a mono-NTA group, a coumarin fluorophore, four-carbon connecting chain, and an arylazide incorporated into the fluorophore (Lai et al., 2015). Integration of photoactive crosslinker, arylazide, is essential not only for strengthening the protein-binding but also for fluorescent enhancement after UV-irradiation. The second and third generations of probes, **Ni-*NTA-AF*** and **Ni-*NTA-AB***, were developed by incorporating fluorophores of fluorescein and BODIPY, respectively. It has been demonstrated that these probes could light up His_6_-tagged proteins in bacterial cells, mammalian cells and even tobacco leaves. Both green (**Ni-*NTA-AF*)** and red (**Ni-*NTA-AB*)** probes could overcome the cellular intrinsic autofluorescence with better contrast than the blue probe, **Ni-*NTA-AC***. Moreover, these probes can be used to quantify His_6_-tagged proteins with a wider linear range (25–1,000 ng) compared with Western Blot (Chao et al., 2017; Yang et al., 2019). The same strategy was followed by an independent group in Denmark, and they recently reported imidazole carbamate probes to label His-tagged proteins (Mortensen et al., 2019) (Figure 2B).

### Exploring Metalloproteomes

The importance of metals in biology is notable, mostly because metals can play critical roles in life processes, especially acting as the catalytic or structural cofactors (Hoppert, 2011). Metals are also frequently incorporated into pharmaceuticals for diagnostic and therapeutic purposes. Given the complexity of metal-protein interactions, tracking, and mining metalloproteins *in vivo* are great challenges. As a metal chelator, NTA can chelate a wide range of transition metal ions (Nancharaiah et al., 2006). Thus, the application of **Ni-*NTA-AC*** could be further extended. By replacing Ni^2+^ with other metal ions, such as Fe^3+^, Cu^2+^, and Bi^3+^, we designed a metal tunable fluorescence probe, **M-*TRACER*** (Tunable Reagent of Arylazide-Conjugated fluorescER), where M stands for metals (Lai et al., 2017). Incubating cells with the **M-*TRACER***, the probe enters live cells and binds metal-associated proteins. After UV activation, those bound proteins are anchored to the probe via covalent bonds, enabling downstream analysis. Cell lysates from these stained cells were subjected to the combination of 2-D gel for protein separation and matrix-assisted laser desorption/ionization time of flight mass spectrometry (MALDI-TOF-MS) for protein identification. The identified proteins were then subjected to Gene Ontology (GO) enrichment analysis and mapped into protein networks. This approach implements the exploration of endogenous metal-associated proteins in various biological systems, including those proteins that bind metal ions weakly or transiently (Figure 2C).

The specific recognition of metal-associated proteins by the probe was demonstrated by using **Fe-*TRACER*** and human serum transferrin as a showcase. The x-ray structure of the protein adduct shows that **Fe-*TRACER*** binds to the specific ferric iron binding site in the C-lobe of transferrin, with Fe^3+^ coordinating to tyrosine 188 (Y188), while asparagic acid 63 (D63), tyrosine 95 (Y95), and lysine 206 (K206) participate in the formation of H-bonding to stabilize the structure. Interestingly, the probe folds a sandwich-like structure and Fe^3+^ coordinates to only two carboxylates and one nitrogen of the probe and one oxygen from Y188, leaving enough vacant site for interacting with proteins (Figure 2D).

Using **Ni-*TRACER***, Ni-associated proteome in *Helicobacter pylori* (*H. pylori*) was firstly mined and 44 Ni^2+^-associated proteins were identified (Lai et al., 2017). Similarly, 63 Bi^3+^-binding proteins were identified by **Bi-*TRACER***, in combination with quantitative proteomics. Then totally 119 Bi-regulated proteins were identified (Wang et al., 2017), providing rich resources to understand the mechanism of bismuth drugs, though these drugs have been applied to treat *H. pylori* infection clinically for decades (Gerrits et al., 2006) (Figure 2E). To deeply understand the metal homeostasis in pathogens, e.g., iron in *Porphyromonas gingivalis* (*P. gingivalis*), a keystone species to trigger periodontitis (Hajishengallis et al., 2012), **Fe-*TRACER*** was employed to unveil Fe-associated proteome in *P. gingivalis*, for this kind of bacteria is highly iron-dependent with a full arsenal of heme-binding proteins (Ciuraszkiewicz et al., 2014; Hajishengallis, 2015). Finally, 17 Fe-associated proteins were identified and they were mostly enriched in 8 biological processes and 2 molecular functions (Jiang et al., 2018). Similarly, the endogenous metalloproteome in live eukaryote cells can be tracked and mined by this approach. In total 54 Cu-associated proteins were identified in HeLa cells by **Cu-*TRACER***, with one-third being confirmed previously by IMAC (Lai et al., 2017).

## Molecular Modifications and Delivery Majorization

### Development of Fluorophores

The development of delocalized electronic structure, i.e., the π system, just leads to the evaluation of fluorophores from blue to red fluorescence for the energy matching with π/π^*^ energy gap finally resulted in fluorescent emission. Among numerous blue fluorophores, the coumarins appeared to be the oldest ones (Kumar et al., 2015). Their easy synthesis, photostability, and large Stokes shift have made our minds to select a coumarin derivative in the design of ***NTA-AC***. Fluoresciens are the most recognized fluorophores with green emissions, which means a collection of available synthetic routes (Fu and Finney, 2018). ***NTA-AF*** incorporated a fluorescein fluorophore with the arylazide group for a significant turn-on effect after UV-activation. Rhodamines and BODIPYs act as the red fluorophores. The latter ones are comparatively new and the rigidity of BODIPYs limits their vibrational state density (Fu and Finney, 2018). Such a limitation endows BODIPYSs with sharp absorption and emission bands, matching with the design of ***NTA-AB***. The use of near infra-red (NIR) fluorophores became a fast-growing and exciting field because of low absorption through biological matrixes (Sevick-Muraca, 2012). A series of NIR fluorescent nanoparticles were designed based upon benzo[1, 2-b: 4, 5-b']dithiophene 1, 1, 5, 5-tetraoxide (BDTO), and they showed good photostability and biocompatibility in cells and animals (Zhen et al., 2018). Imaging at the second near-infrared window (NIR-II; 1,000–1,700 nm) has also been achieved, and antiquenching cyanine fluorophores were reported by providing the optical penetration as deep as 8 mm with high contrast and super photostability (Wang et al., 2019). Herein, the development of NIR probes is urgently required to visualize metalloproteins.

### Delivery of Probes Into Cells

To track intracellular POI, the delivery of fluorescent probes appeared to be a critical aspect. To break the limitations of lipophilic cell membrane toward large, charged molecules, about five kinds of approaches have been reported for living cell imaging (Lymperopoulos et al., 2010). Among them, electroporation enabled a high efficiency of fluorophores and proteins with notable size (up to 60 kDa) into living *E. coli* cells. Even though high voltages certainly decrease the cell viability, researchers can select higher ionic strengths with lower electroporation voltages and use glycerol to slightly increase the threshold of suitable ionic strength (Sustarsic et al., 2014). Addition of detergents, polyamidoamine and peptide dendrimers can be available for both prokaryotic and eukaryotic cells whilst bacteria usually required a larger amount. Tween 80 (0.25%) assisted **Ni-*NTA-AB*** to label intracellular His_6_-tagged protein whilst we found no significant effect of Tween 80 on cell viability (Chao et al., 2017). Besides these, arginine-rich CPPs have been demonstrated to induce multilamellarity of the cell membrane and subsequently enter cells via the formation of a fusion pore (Allolio et al., 2018). The design of a CPP carrier successfully improved the membrane permeability of Ni-based probes in mammalian cells (Uchinomiya et al., 2013). The last two approaches were microinjection and use of pore-forming agents such as streptolysin-O (SLO). In particular, SLO, a bacterial toxin, can form temporary pores in the membrane for a wide range of probes including small ligands. The efficiency achieved as high as 85% while around 95% of cells were reported to contain intact membrane after recovery of SLO treatments (Teng et al., 2016).

## Concluding Remarks

Two families of representative probes based on metal-oriented protein recognition, i.e., As(III)-based probes and metal-NTA-based probes, were introduced and summarized from their binding mechanisms, molecule structures, to biological applications. These probes initially targeted fused proteins with either tetracysteine or His_6_-tags. For imaging of extracellular and intracellular proteins, almost all As(III)-based probes entered cells successfully whilst an array of Ni-NTA-based probes lack cellular membrane permeability. Despite diverse structure optimizations on these Ni-NTA-based probes, no clear clue of cellular membrane permeability was presented. Consequently, the different permeability between these two classes of probes resulted in their different applications in biology besides labeling relevant peptide-fused proteins. As(III)-based probes light up the native As(III)-binding proteins (Huang et al., 2011) while the proteomic analysis is achieved by the combination of the microarray with Biotin-As (Zhang et al., 2015). For the Ni-NTA-based probes, the Ni^2+^-binding proteins are detected in cell lysates and membrane parts (Kapanidis et al., 2001; Guignet et al., 2004; Arnau et al., 2006; Soh, 2008; Jing and Cornish, 2011).

Our probes were built upon metal-NTA coordination chemistry, and Ni^2+^ was selected as a showcase to inherit the success of Ni-NTA-based probes. In total, three probes were synthesized, i.e., **Ni-*NTA-AC***, **Ni-*NTA-AF***, and **Ni-*NTA-AB***, with fluorescent emission ranging from blue to red. Importantly, they can track intracellular His-tagged proteins in live cells (Lai et al., 2015; Chao et al., 2017). The multiple-metal-binding ability of NTA expands the toolbox for these probes. Chelating with a variety of transition metals, the probes (**M-*TRACER***) can label the relevant metal-associated proteins through metal ion-oriented recognition. As a result, the networks of Ni^2+^, Bi^3+^, Cu^2+^, and Fe^2+/3+^-associated proteins (and their proteomes) have been lit up *in situ* and *in vivo* (Lai et al., 2017; Wang et al., 2017; Jiang et al., 2018; Li et al., 2018). Subsequent protein identification and function analysis can be achieved by proteomics and other omics techniques. More sensitive and selective probes will be generated from further modifications of fluorophores, as well as optimizations of molecule delivery. They may provide a new horizon to understand bacterial and human metalloproteomes, and their relationship with human health such as infectious disease and drug development.

## Author Contributions

NJ drafted this manuscript. HL revised this whilst. HS supervised this.

### Conflict of Interest Statement

The authors declare that the research was conducted in the absence of any commercial or financial relationships that could be construed as a potential conflict of interest.
